# Ultrastructural and Immunohistochemical Study on the Nephrotoxicity Following Intravitreal Administration of the Antifungal Agents Voriconazole and Micafungin in New Zealand White Rabbits

**DOI:** 10.3390/ijms262010129

**Published:** 2025-10-17

**Authors:** Sofia Karachrysafi, Vasileios-Alexandros Karakousis, Alexandros Liatsos, Dimitrios Kavvadas, Despoina Ioannou, Pinelopi Anastasiadou, Evangelia Kofidou, Vasileios Karampatakis, Antonia Sioga, Nikolaos Raikos, Theodora Papamitsou

**Affiliations:** 1 Research Team “Histologistas”, Interinstitutional Postgraduate Program “Health and Environmental Factors”, Department of Medicine, Faculty of Health Sciences, Aristotle University of Thessaloniki, 54124 Thessaloniki, Greece; alexanderkarakousis@gmail.com (V.-A.K.); aliatsob@auth.gr (A.L.); kavvadas@auth.gr (D.K.); dioana@auth.gr (D.I.); apinelop@dent.auth.gr (P.A.); sioga@auth.gr (A.S.); thpapami@auth.gr (T.P.); 2 Laboratory of Histology-Embryology, School of Medicine, Faculty of Health Sciences, Aristotle University of Thessaloniki, 54124 Thessaloniki, Greece; 3 Department of Oral Pathology and Surgery, Laboratory of Stomatology, Department of Dentistry, Aristotle University of Thessaloniki, 54124 Thessaloniki, Greece; 4 Veterinary School, School of Health Sciences, Aristotle University of Thessaloniki, 54124 Thessaloniki, Greece; evikofidou@gmail.com; 5 Laboratory of Experimental Ophthalmology, School of Health Sciences, Department of Medicine, Aristotle University of Thessaloniki, 54124 Thessaloniki, Greece; karopthth@auth.gr; 6 Laboratory of Forensic Medicine and Toxicology, School of Medicine, Faculty of Health Sciences, Aristotle University of Thessaloniki, 54124 Thessaloniki, Greece; raikos@auth.gr

**Keywords:** nephrotoxicity, ultramicroscopic study, immunohistochemical study, EGFR, IL-6, intravitreal administration, antifungal agents, voriconazole, micafungin, proximal tubules

## Abstract

The aim of the present study is to examine the possibility of nephrotoxicity following the intravitreal injection of the antifungal agents voriconazole and micafungin. Μale and female New Zealand white rabbits were divided into the control group C that received no medication and the study groups that underwent either one or two intravitreal injections of voriconazole or micafungin solution, respectively, or one co-administration of the two agents. Euthanasia was performed ten days after the last intravitreal administration, and kidney tissue samples were obtained and prepared for electron microscopy study, as well as immunohistochemical study for EGFR and IL-6 markers. Ultrastructural alterations of the renal tissue were found in places of limited extent, more evident at the level of the proximal tubules. The expression of the two markers was positive, especially in the double and the combined administration of the two drugs, both in the renal corpuscle and the tubules. The finding of the aforementioned histological lesions triggers the need for an additional study of the effect of the specific drugs on the kidney to establish whether these alterations are reversible or not. Redesigning the dosage regimen during intravitreal administration of these agents could be a future therapeutic goal to prevent potential nephrotoxicity. The intravitreal concentrations used in rabbits, particularly for voriconazole, closely approximate those used in humans, supporting the clinical relevance of the findings.

## 1. Introduction

### 1.1. Fungal Endophthalmitis (FE)

Fungal Endophthalmitis (FE) is a rare yet severe intraocular infection that may result in permanent vision loss or blindness [1]. It arises from either exogenous sources, such as ocular trauma, post-surgical complications, or infectious keratitis, or endogenous spread via hematogenous dissemination [2,3]. Exogenous FE is typically unilateral and may present acutely or chronically (lasting more than six weeks) [4]. Although bacterial and fungal agents can both cause endophthalmitis with similar frequency, Candida albicans is the most common fungal species involved, followed by Aspergillus [5,6]. Regional studies highlight the burden of fungal FE; for example, in India, postoperative FE accounted for 46.8% of cases, followed by trauma (35.6%) and endogenous origins (17.5%) [7]. The predominant fungi included *Aspergillus* (39%), *Candida* (15.1%), and *Fusarium* (15.9%) [7]. Other reports confirm that fungal keratitis can progress to endophthalmitis, particularly in the presence of steroid use, aphakia, and large corneal ulcers [8].

Prompt diagnosis and immediate initiation of systemic and intravitreal antifungal therapy are critical [9]. However, treatment remains challenging due to poor ocular tissue penetration and the dose-dependent fungicidal action of antifungals. Commonly used agents include polyenes (e.g., Amphotericin B), azoles (e.g., Voriconazole), and increasingly, echinocandins (e.g., Micafungin, Caspofungin), which show promising results against *Candida* and *Aspergillus* with low toxicity [10].

### 1.2. Voriconazole and Micafungin: Pharmacology and Toxicity Concerns

Voriconazole is an antifungal drug, part of the triazoles. Triazoles are compounds that contain the imidazole or 1,2,4-triazole ring, the presence of which is essential for the molecule’s antifungal activity [11]. In particular, voriconazole constitutes a second-generation triazole antifungal that gained FDA approval in 2002 [12,13,14,15]. Structural modifications, including a fluoropyrimidine ring and α-methyl group, enhanced its potency and target affinity, improving efficacy against *Candida albicans*, *C. glabrata*, and *Aspergillus* spp. [12,16,17].

Voriconazole is available in oral (50–200 mg tablets or 40 mg/mL suspension) and intravenous formulations. Due to its poor aqueous solubility, the IV form includes the solubilizing agent β-cyclodextrin sodium sulfobutyl ether (SBECD), which is excreted renally and does not interfere with the drug’s pharmacokinetics [18]. The oral regimen involves a 400 mg loading dose every 12 h, followed by a 200 mg maintenance dose; the IV dosage is 6 mg/kg every 12 h initially, followed by 4 mg/kg [19,20]. Voriconazole inhibits the CYP450-dependent 14α-demethylation of lanosterol, a critical step in fungal ergosterol synthesis [21,22,23,24]. However, it can also inhibit human CYP enzymes (CYP3, CYP2, CYP1), leading to notable drug interactions [25,26]. Key structural elements like the 3′ methyl group and difluorobenzene enhance its antifungal activity [27]. It has broad-spectrum fungicidal activity, including against fluconazole-resistant Candida strains and amphotericin B-resistant *Aspergillus terreus* [28,29,30]. While it has excellent oral bioavailability and wide clinical use [31,32,33,34,35,36], intravenous voriconazole has been associated with transient visual disturbances, hepatotoxicity, photosensitivity, and, in rare cases, nephrotoxicity, particularly due to the accumulation of the solubilizing agent SBECD in renal impairment [37,38,39,40,41,42,43,44]. Resistance can also develop through CYP51A mutations and efflux mechanisms [45,46,47,48,49].

Micafungin, an echinocandin antifungal, represents the first class targeting the fungal cell wall [50]. These semi-synthetic lipopeptides are derived from natural fermentation: caspofungin from *Glarea lozoyensis*, micafungin from *Aspergillus nidulans* var. echinulatus, and anidulafungin from *Coleophoma empetri* [51,52]. Structurally similar, all are cyclic lipopeptides administered intravenously, with low toxicity and minimal drug interactions [53]. Their activity is attributed to distinct N-linked acyl side chains—fatty acid (caspofungin), aromatic (micafungin), and alkoxytriphenyl (anidulafungin) [52]; homotyrosine residues contribute to enzyme inhibition, while proline residues enhance activity [54]. Micafungin is available in 50 mg and 100 mg vials, with prophylactic (50 mg/day for 20 days) and therapeutic (50–150 mg/day for 15–20 days) regimens [55,56]. Echinocandins inhibit β-(1,3)-D-glucan synthase (FKS1), disrupting cell wall integrity and inducing osmotic lysis [57,58,59,60]. They are fungicidal against Candida, including azole-resistant strains, and fungistatic against Aspergillus [61,62,63,64]. Their antifungal activity against biofilms makes them useful in treating infections related to catheters or prosthetic devices [65]. They are first-line agents for esophageal and invasive candidiasis, especially in neutropenic and stem cell transplant patients [66,67,68].

Pharmacokinetically, micafungin shows high plasma protein binding (~99.5%), poor CNS penetration, and is only effective when administered intravenously [69,70,71,72]. It undergoes partial hepatic metabolism, with limited CYP450 involvement [73,74,75], and its long half-life supports once-daily dosing [76,77]. Toxicity is generally low, with rare gastrointestinal symptoms, liver enzyme alterations, and histamine-mediated reactions [78,79,80]. Caspofungin shows low nephrotoxicity, though in vitro studies suggest potential tubular damage [81,82]. Micafungin may mildly elevate the levels of immunosuppressants, but serious renal toxicity is rarely observed in clinical settings [83,84,85]. Resistance, particularly in *C. glabrata*, is linked to FKS gene mutations and is associated with prior echinocandin use. This resistance is increasing globally and often co-occurs with azole resistance, worsening outcomes [86,87,88].

Administered drugs—Voriconazole and Micafungin—properties are presented in Table 1.

### 1.3. Intravitreal Administration: A Local Route of Administration?

The removal of the medicinal substance from the vitreous fluid after its intravitreal injection is carried out by its initial biotransformation in the ocular tissues and its final removal from the ocular compartments into the systemic circulation.

**Figure 1:**
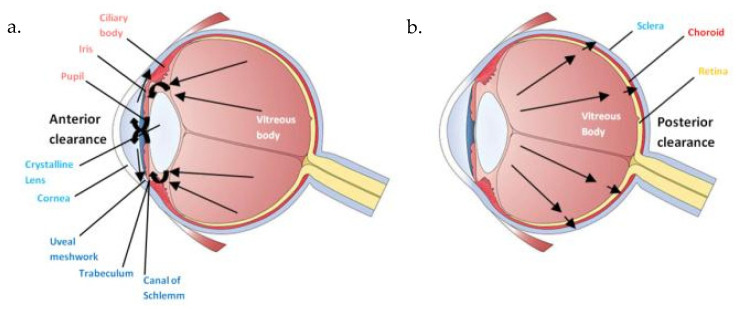
Intravitreal administration pharmacokinetics. (**a**) Anterior clearance; (**b**) Posterior Clearance.

Intravitreal injection is primarily considered a local drug delivery route. However, systemic absorption can occur via anterior and posterior clearance pathways [89,90,91,92,93]. In anterior clearance, drugs diffuse through the aqueous humor into the systemic circulation, while in posterior clearance, small lipophilic molecules such as voriconazole cross the retina and enter the choroidal vasculature [94,95,96,97,98,99]. Thus, even local administration can result in systemic exposure. (Figure 1)

This has been demonstrated for intravitreal anti-VEGF agents, which, despite ocular delivery, reduce systemic VEGF activity and contribute to hypertension, proteinuria, and chronic kidney disease [99,100]. Therefore, it is essential to consider the potential systemic effects—including nephrotoxicity—of antifungals delivered intravitreally.

### 1.4. Renal Vulnerability

**Figure 2:**
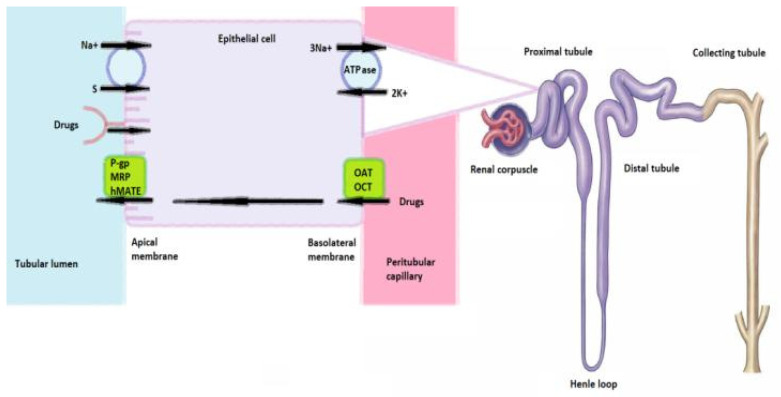
Mechanisms of proximal renal tubules’ vulnerability to xenobiotics.

Proximal tubular cells are particularly susceptible to drug-induced toxicity due to their active reabsorptive and secretory roles and exposure to high concentrations of circulating xenobiotics, as the kidneys receive approximately 25% of cardiac output. Drugs reach these cells via basolateral transporters (OATs, OCTs) and apical pathways, including P-glycoprotein, MRPs, hMATE1, endocytosis, and sodium co-transporters dependent on Na^+^/K^+^-ATPase gradients. (Figure 2) Intracellular metabolism may yield toxic intermediates, while mitochondrial impairment and ROS generation can lead to acute tubular necrosis [101,102,103,104].

Renal effects of voriconazole and micafungin remain underexplored. In vivo studies by Somchit et al. (2012) [42] showed no significant serum biochemical changes following voriconazole exposure in rats, though mild histological inflammation and ultrastructural alterations were observed. Degrimenci et al. (2019) [105] similarly reported epithelial degeneration and inflammation. While echinocandins, including micafungin, are generally non-nephrotoxic, even at high doses [80], isolated clinical reports suggest rare renal involvement.

Voriconazole, though safer than amphotericin B, has been linked to AKI, interstitial nephritis, and elevated serum creatinine, particularly in at-risk populations. These effects are typically reversible upon discontinuation or transition to oral therapy. Micafungin is generally well tolerated; however, long-term animal studies have noted reversible renal tubular vacuolization and bladder epithelial hyperplasia [104]. Routine renal function monitoring is advised to ensure therapeutic efficacy and minimize toxicity.

### 1.5. Immunohistochemical Markers

IL-6 is a 184-amino acid glycoprotein produced by various immune and non-immune cells, including T and B lymphocytes, macrophages, endothelial cells, and fibroblasts [106]. Initially identified as a regulator of B-cell and Th17 differentiation [107], IL-6 is now recognized as a pleiotropic cytokine involved in hematopoiesis, angiogenesis, immune regulation, inflammation, wound healing, and tumorigenesis across several malignancies (e.g., lymphoma, multiple myeloma, and carcinomas of the lung, breast, prostate, and kidney) [108]. It displays both pro- and anti-inflammatory effects via two distinct signaling pathways: classical signaling, which mediates protective responses, and trans-signaling, implicated in chronic inflammation and disease pathogenesis [109,110,111,112,113]. IL-6 overproduction is linked to autoimmune, fibrotic, and neoplastic conditions, while its deficiency impairs tissue repair processes [114]. IL-6 immunohistochemistry has been applied in diverse models, revealing roles in subretinal fibrosis [115], keratoconjunctivitis [116], and renal pathology. In the kidney, IL-6 is upregulated post-injury, promotes endothelin-1 production, and contributes to hypertension, fibrosis, and chronic kidney disease progression, possibly exerting autocrine effects in renal carcinogenesis [117,118].

EGFR (HER1), a 170 kDa transmembrane glycoprotein primarily expressed in the kidney and salivary glands [119], possesses intrinsic tyrosine kinase activity. Ligand binding (e.g., EGF, TGF-α, HB-EGF) triggers receptor dimerization, activating downstream pathways—JAK/STAT, RAS/MAPK, and PI3K/AKT/mTOR—regulating proliferation, survival, angiogenesis, and metastasis [120,121]. Aberrant EGFR signaling promotes tumorigenesis, therapeutic resistance, and poor prognosis in multiple epithelial cancers, making it a key therapeutic target in several solid malignancies [122].

EGFR antibodies are widely used in immunohistochemistry [123], with applications ranging from ocular pathophysiology (e.g., myopia) [124] to renal studies. EGFR is predominantly expressed in renal tubular cells and to a lesser extent in glomerular cells, making it a valuable marker in nephropathology. EGFR dysregulation in kidney diseases has been linked to epithelial hyperplasia, cyst formation, and fibrosis, mimicking polycystic kidney disease [125,126]. Moreover, EGFR-targeted therapies in animal models have shown potential in managing PKD and renal fibrosis [127], while EGFR antagonists have been associated with drug-induced nephrotoxicity [128].

### 1.6. Aim of This Study

This study examines the potential nephrotoxic effects of intravitreal voriconazole and micafungin, used for fungal endophthalmitis [129,130]. New Zealand white rabbits received intravitreal injections, and renal tissue was analyzed via light and electron microscopy to identify structural alterations consistent with known nephrotoxicity [131,132]. Despite limited data on renal effects after intravitreal administration and no prior in vivo rabbit comparisons, findings revealed microscopic and ultrastructural renal lesions, indicating that systemic absorption—potentially up to 50%—occurs, exposing the kidneys [133,134,135,136].

## 2. Results

The results following the applied interventions are presented in this section. In control group C, no alteration in the architecture of the renal tissue was observed in any of the applied interventions.

### 2.1. Light Microscopy-Immunohistochemistry Results

**Figure 3:**
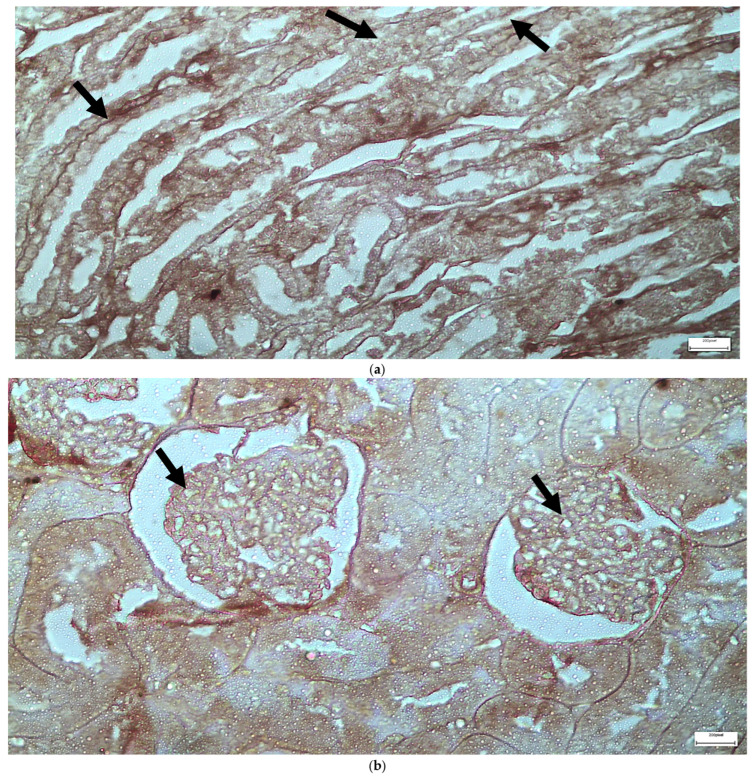
(**a**,**b**) Immunohistochemical expression of IL-6 with mild (+) intensity in the cells of the wall of the renal tubules [↑] in the renal medulla (**a**) and in the cells of the renal corpuscles [↑] in the renal cortex (**b**) after one intravitreal injection of voriconazole (Subgroup V1). ×400.

**Figure 4:**
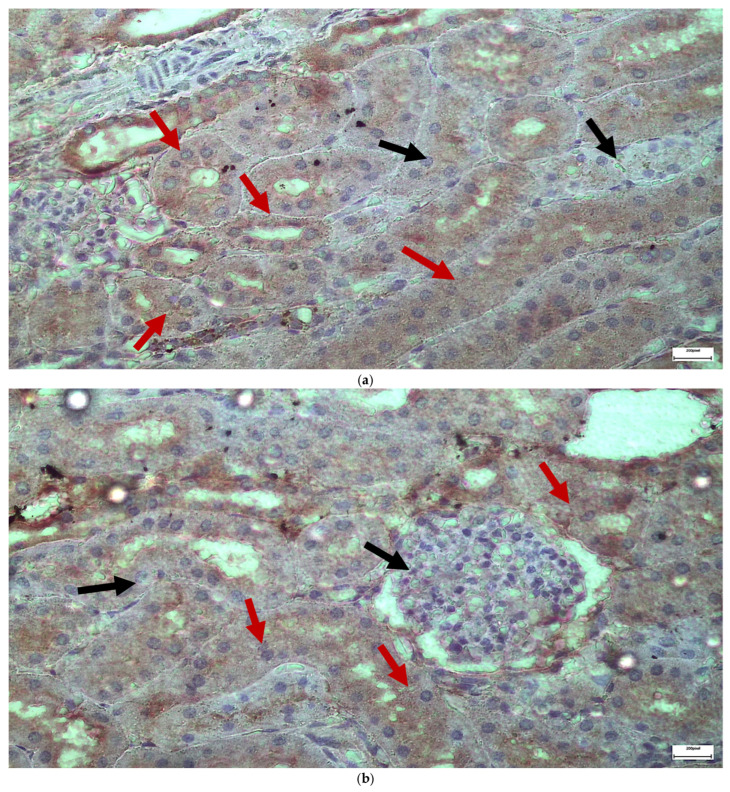
(**a**,**b**) Immunohistochemical expression of EGFR with mild (+) [↑] to moderate (++) [↑] intensity in the cells of the wall of the renal tubules in the renal medulla (**a**) and in the cells of the renal corpuscles in the renal cortex (**b**) after one intravitreal injection of voriconazole (Subgroup V1). ×400.

**Figure 5:**
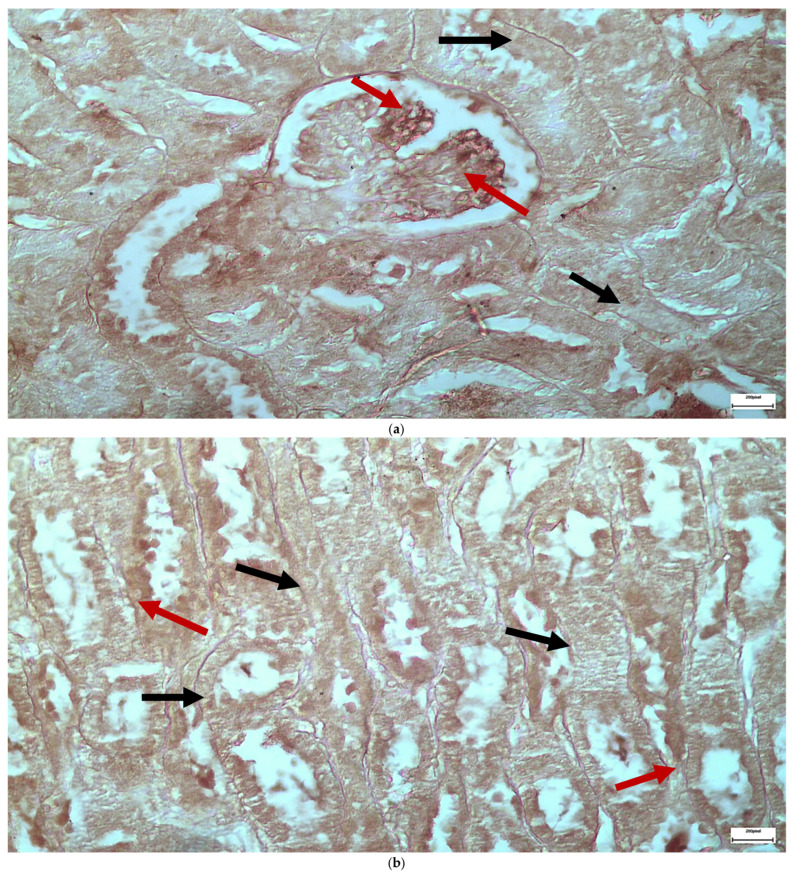
(**a**,**b**) Immunohistochemical expression of IL-6 with mild (+) [↑] to moderate (++) [↑] intensity after two intravitreal injections of voriconazole. Moderate intensity of IL-6 expression is observed in renal corpuscles (**a**), while mild and in some places moderate intensity is observed in renal tubules (**b**). ×400.

**Figure 6:**
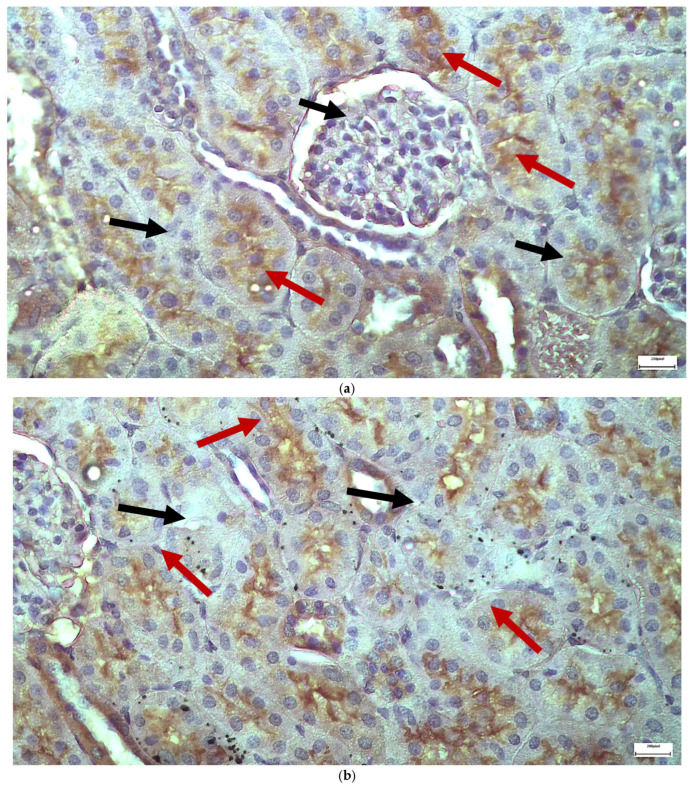
(**a**,**b**) Immunohistochemical expression of EGFR with mild (+) [↑] to moderate (++) [↑] intensity after two intravitreal injections of voriconazole. Mild intensity is observed in renal corpuscles (**a**), while mild to moderate intensity of IL-6 expression is observed in renal tubules (**a**,**b**). ×400.

**Figure 7:**
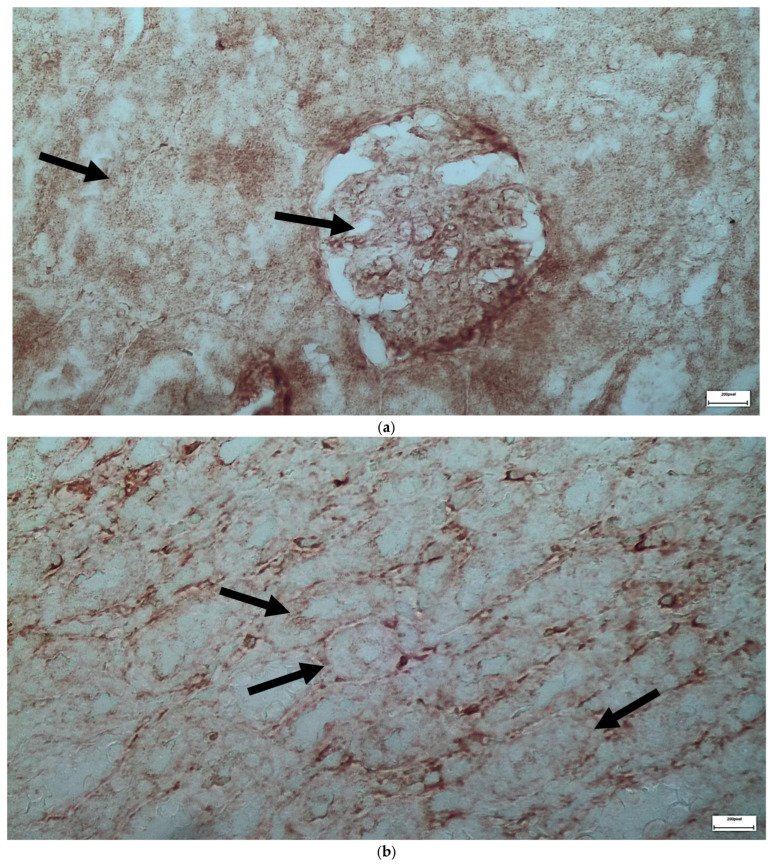
(**a**,**b**) Immunohistochemical expression of IL-6 with mild (+) [↑] intensity after one intravitreal injection of micafungin, both in the renal corpuscles (**a**) and the renal tubules (**b**). ×400.

**Figure 8:**
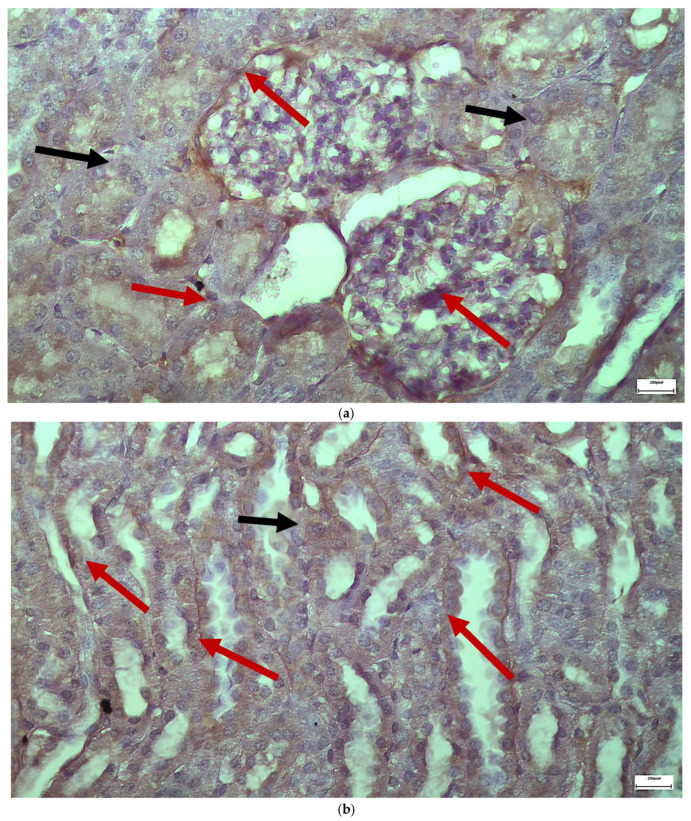
(**a**,**b**) Immunohistochemical expression of EGFR with mild (+) [↑] to moderate (++) [↑] intensity after one intravitreal injection of micafungin, both in the renal corpuscles (**a**) and the renal tubules (**b**). ×400.

**Figure 9:**
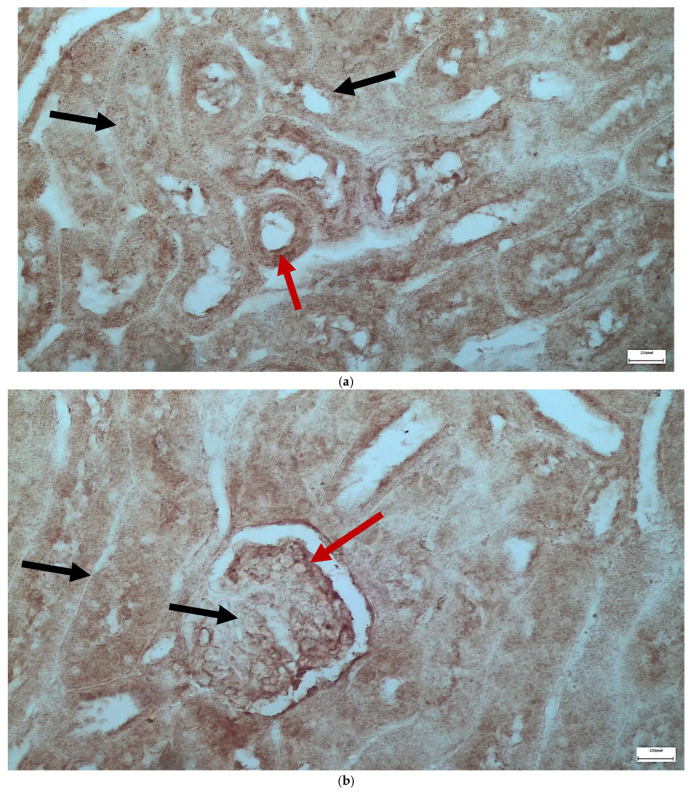
(**a**,**b**) Immunohistochemical expression of IL-6 with mostly mild (+) [↑] to some places moderate (++) [↑] intensity after two intravitreal injections of micafungin in the renal corpuscles and the renal tubules. ×400.

**Figure 10:**
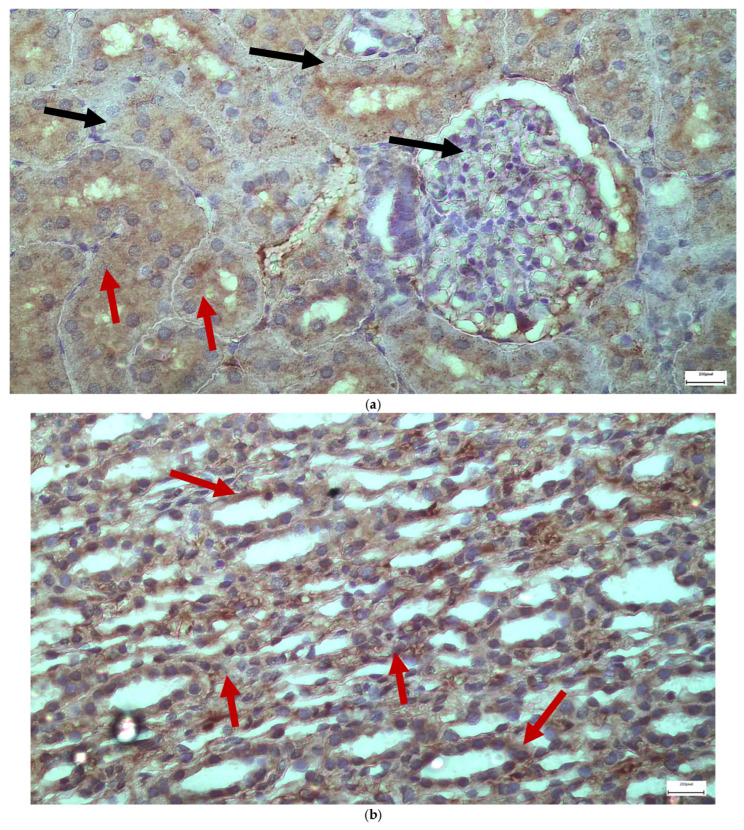
(**a**,**b**) Immunohistochemical expression of EGFR with mild (+) [↑] to moderate (++) [↑] intensity after two intravitreal injections of micafungin. Renal tubules demonstrate mostly moderate intensity of EGFR in contrast to the renal corpuscles. ×400.

**Figure 11:**
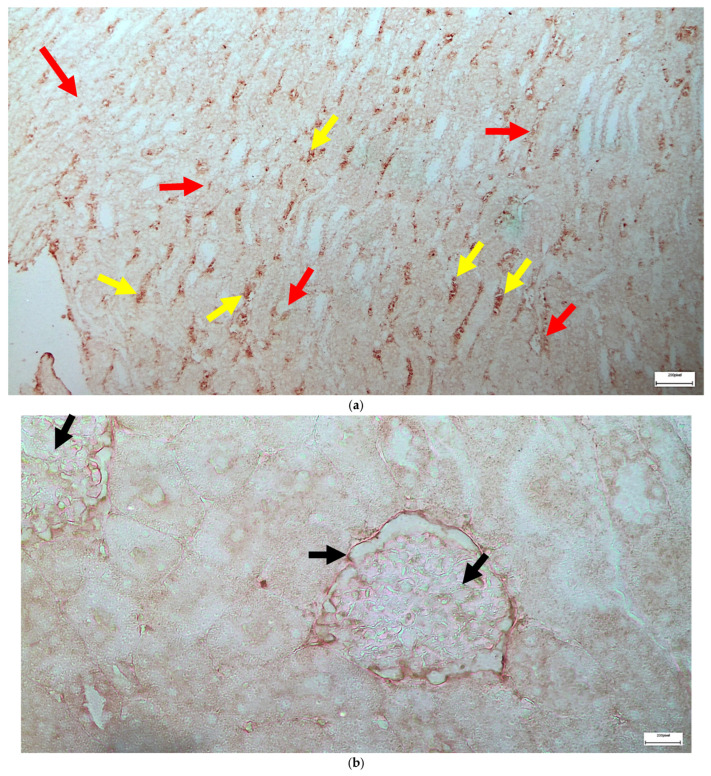
(**a**,**b**) Immunohistochemical expression of IL-6 with mostly moderate (++) [↑] to strong (+++) [↑] intensity in renal tubules in the renal medulla (**a**) and with mild (+) [↑] intensity in renal corpuscles (**b**) after simultaneous intravitreal injection of voriconazole and micafungin. ×100 (**a**), ×400 (**b**).

**Figure 12:**
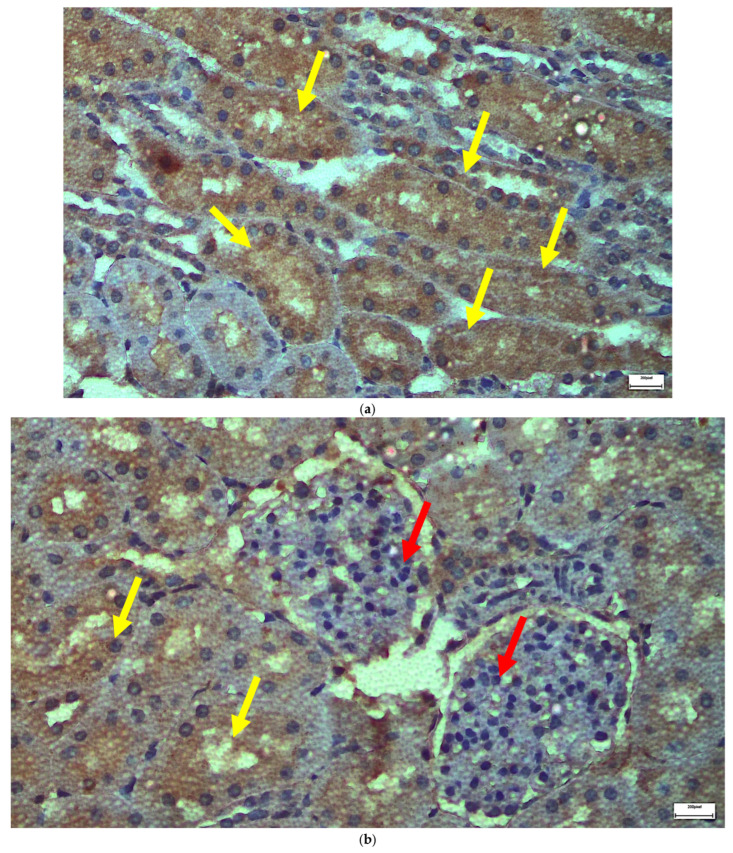
(**a**,**b**) Immunohistochemical expression of EGFR with strong (+++) [↑] intensity in renal tubules (**a**,**b**) and with moderate (++) [↑] intensity in renal corpuscles (**b**) after simultaneous intravitreal injection of voriconazole and micafungin. ×400.

The immunohistochemical staining results reveal that both IL-6 and EGFR expression levels varied depending on the antifungal treatment and dosage. Single (Figure 3 and Figure 4) and double (Figure 5 and Figure 6) doses of voriconazole generally produced mild (+) to moderate (++) staining intensities for IL-6 and EGFR. In a similar way, single (Figure 7 and Figure 8) and double (Figure 9 and Figure 10) doses of micafungin generally produced mild (+) to moderate (++) staining intensities for IL-6 and EGFR. Notably, the combination of voriconazole and micafungin (VM) resulted in the highest staining intensities, with IL-6 (Figure 11) showing moderate (++) to strong (+++) staining in renal tubules and EGFR (Figure 12) exhibiting strong (+++) staining in renal tubules and moderate (++) staining in renal corpuscles. These findings suggest a dose-dependent and synergistic effect of the combined antifungal treatment on inflammatory and growth factor markers in renal tissues.

**Figure 13:**
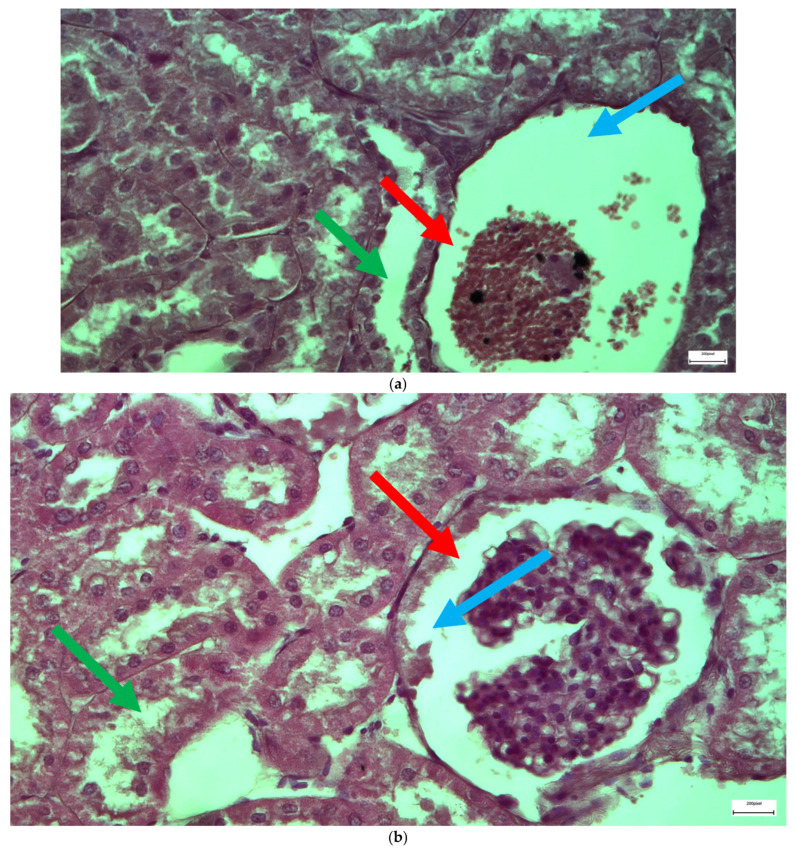
(**a**,**b**) Following the administration of a single dose of voriconazole or micafungin, alteration of the architecture of the renal glomeruli and thickening of the vascular glomerulus (↑), was observed along with enlargement of the urinary cavity (↑), and dilation of the renal tubules (↑), more prominent in the level of collecting tubules whose architecture has been greatly disrupted. ×400.

**Figure 14:**
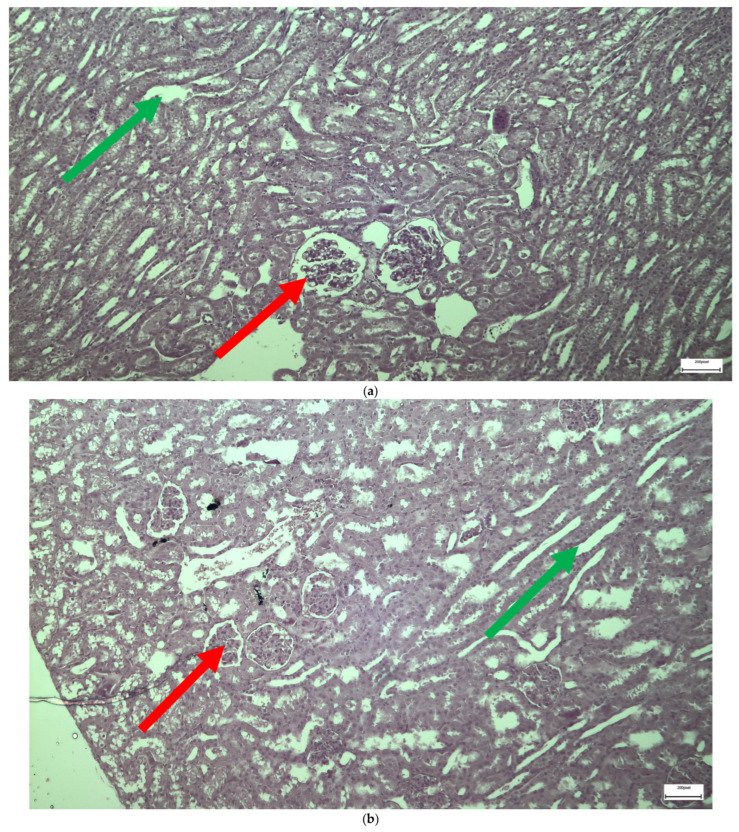
(**a**,**b**) Administration of a double dose of voriconazole results in local thickening with shrinkage of the renal glomeruli (↑), as well as the dilation of the renal tubules (↑), expressed as a disturbance of the normal architecture of the wall, and to a greater extent, compared to the single dose injection of the abovementioned agents. ×100.

**Figure 15:**
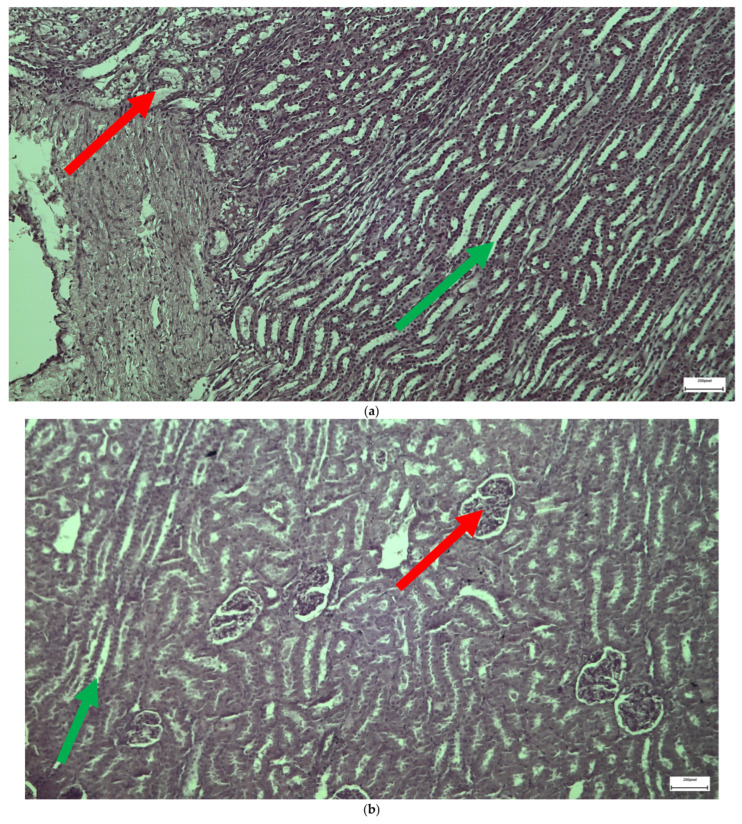
(**a**,**b**) Administration of a double dose of micafungin results in local thickening with shrinkage of the renal glomeruli (↑), as well as the dilation of the renal tubules (↑), expressed as a disturbance of the normal architecture of the wall, and to a greater extent, compared to the single dose injection of the abovementioned agents. ×100.

**Figure 16:**
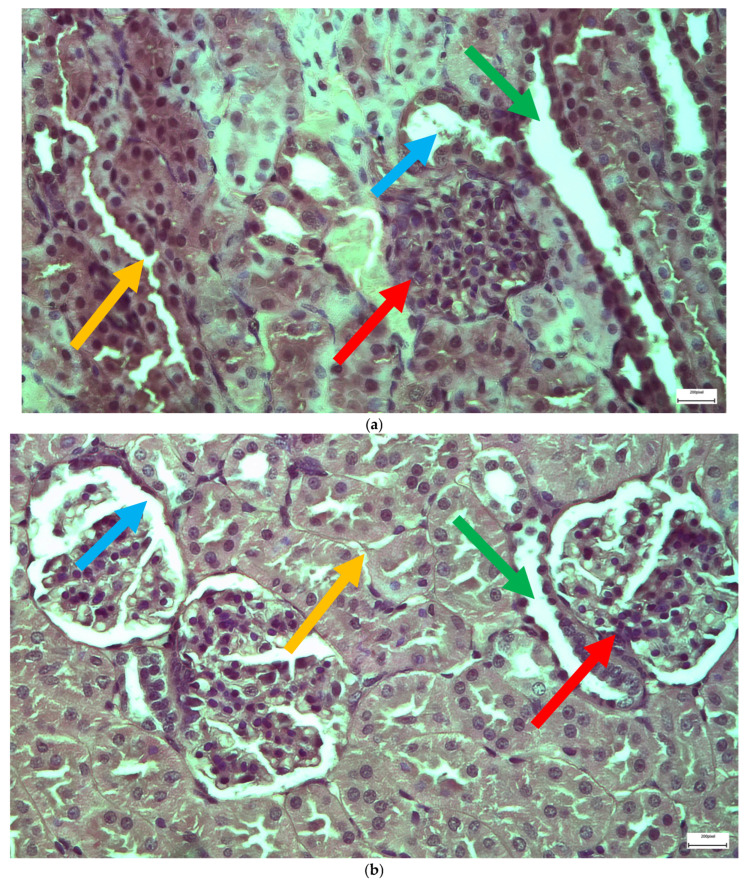
(**a**,**b**) After the simultaneous infusion of voriconazole and micafungin, histologically enlarged spaces in the form of intercellular edema (↑) are observed in some places in the level of the connective tissue, together with enlargement of the urinary cavity (↑), shrinkage of the vascular glomerulus and the renal glomerulus (↑), and the dilation of the renal tubules (↑). ×400.

The single dose of voriconazole or micafungin leads to noticeable alteration of the renal glomerular architecture and thickening of the vascular glomerulus (Figure 13). These changes are accompanied by enlargement of the urinary cavity and dilation of renal tubules, particularly at the level of the collecting tubules, where significant structural disruption is observed. The effects of a double dose of voriconazole (Figure 14) cause local thickening and shrinkage of the renal glomeruli, along with marked dilation of the renal tubules. The extent of these changes reflects a greater disturbance of the normal renal architecture compared to single-dose administration. The double dose of micafungin (Figure 15) results in comparable shrinkage and thickening of the glomeruli, as well as pronounced tubule dilation, again more severe than that observed with a single dose, indicating a dose-dependent nephrotoxic effect. Moving on to the simultaneous infusion of voriconazole and micafungin (Figure 16), renal tissue reveals intercellular edema within the connective tissue, enlargement of the urinary cavity, shrinkage of both the vascular and renal glomeruli, and significant dilation of the renal tubules. These findings suggest a synergistic or cumulative renal impact when both agents are administered together.

### 2.2. Statistical Analysis

Immunohistochemical staining for IL-6 and EGFR was assessed in renal tissue sections using the ImmunoReactive Score (IRS) method, which combines staining intensity and the percentage of positive cells to produce a semi-quantitative score. Staining intensity was scored from 0 to 3, while the percentage of positive cells was scored from 0 to 4, with the final IRS calculated by multiplying these values. Scoring was performed independently by two observers to ensure consistency. The results are summarized in Table 2.

For IL-6 expression in renal tubules, a significant difference among the groups was observed (Kruskal-Wallis test, *p* = 0.048). Post hoc analysis revealed that IL-6 levels were significantly elevated in the VM group compared to the control group (*p* = 0.03). No other intergroup differences in tubules reached statistical significance. In renal corpuscles, IL-6 expression also varied significantly across groups (*p* = 0.0089). Expression levels were significantly higher in the VM group (*p* = 0.006) and the M group (*p* = 0.049) when compared with the control group. The V group did not differ significantly from the control in either tubules or corpuscles.

EGFR expression in renal tubules showed significant differences among the experimental groups (*p* = 0.0197). Dunn’s test indicated significantly increased expression in the VM group (*p* = 0.012) and in the V group (*p* = 0.043) compared to controls. The M group did not show a statistically significant difference in this structure. In renal corpuscles, EGFR expression also differed significantly between groups (*p* = 0.0247). Elevated expression was observed in the VM group (*p* = 0.013) and the M group (*p* = 0.038) compared to the control group. No significant difference was detected between the V and control groups in this tissue structure. The results are summarized in Table 3.

The relationship between IL-6 and EGFR expression within the same tissue type was examined using the Chi-square test of independence, applied separately within each experimental group. In the VM group, a statistically significant association was found between IL-6 and EGFR expression in both tubules and corpuscles (*p* < 0.05), suggesting concurrent elevation of both markers. No significant relationship was found in the control group (*p* > 0.05), nor in the V or M groups.

To explore differences in expression between renal tubules and corpuscles for each marker, the Chi-square test of independence was again employed. For IL-6, the analysis yielded a *p*-value above the threshold for significance (*p* > 0.05), indicating similar expression in both tissue structures. In contrast, EGFR expression differed significantly between tubules and corpuscles (*p* < 0.05), with more intense expression observed in corpuscles, particularly in the VM and M groups.

Taken together, these findings indicate that treatment with voriconazole, micafungin, and especially their combination alters the immunohistochemical expression of IL-6 and EGFR in renal tissues. The most pronounced changes occurred in the VM group, with significant elevations in both markers and a coordinated pattern of expression. Moreover, EGFR exhibited structure-specific expression patterns, with higher levels in renal corpuscles.

### 2.3. Electron Microscopy Results

**Figure 17:**
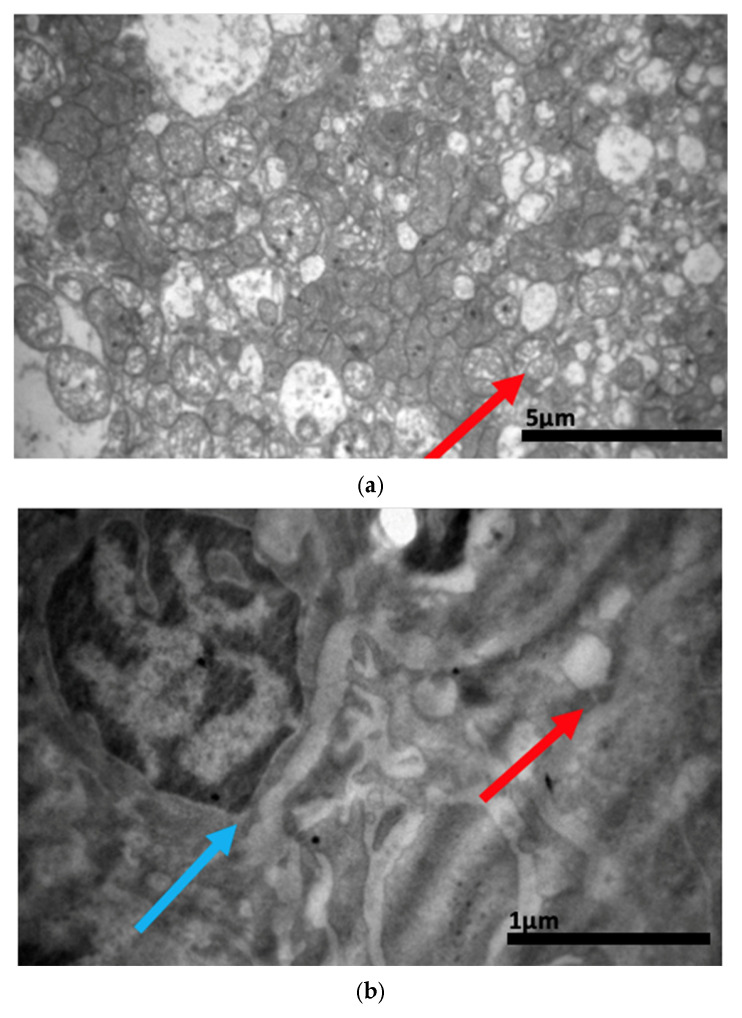

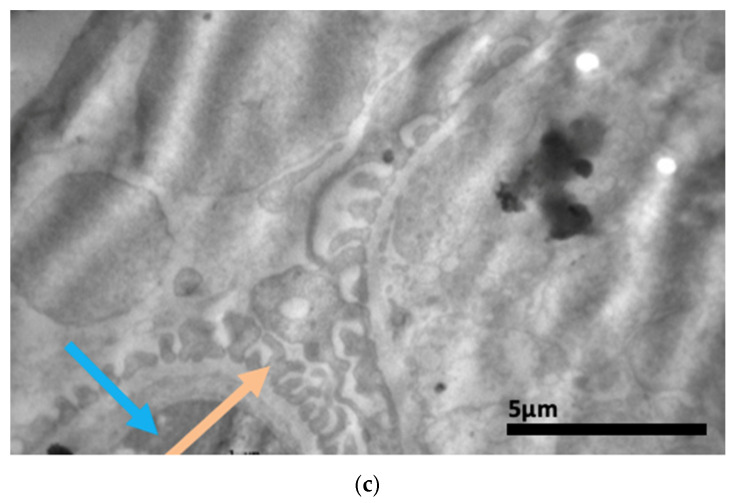
(**a**,**b**,**c**) Following the administration of a single dose of voriconazole, vacuolar degeneration is evident with the appearance of small vacuoles within the proximal convoluted tubule cells (↑). Focal fusion of the podocytes’ pedicels (↑) of Bowman’s capsule and the enlargement of the urinary cavity were also observed (↑). ×8000 (**a**), ×10,000 (**b**,**c**).

**Figure 18:**
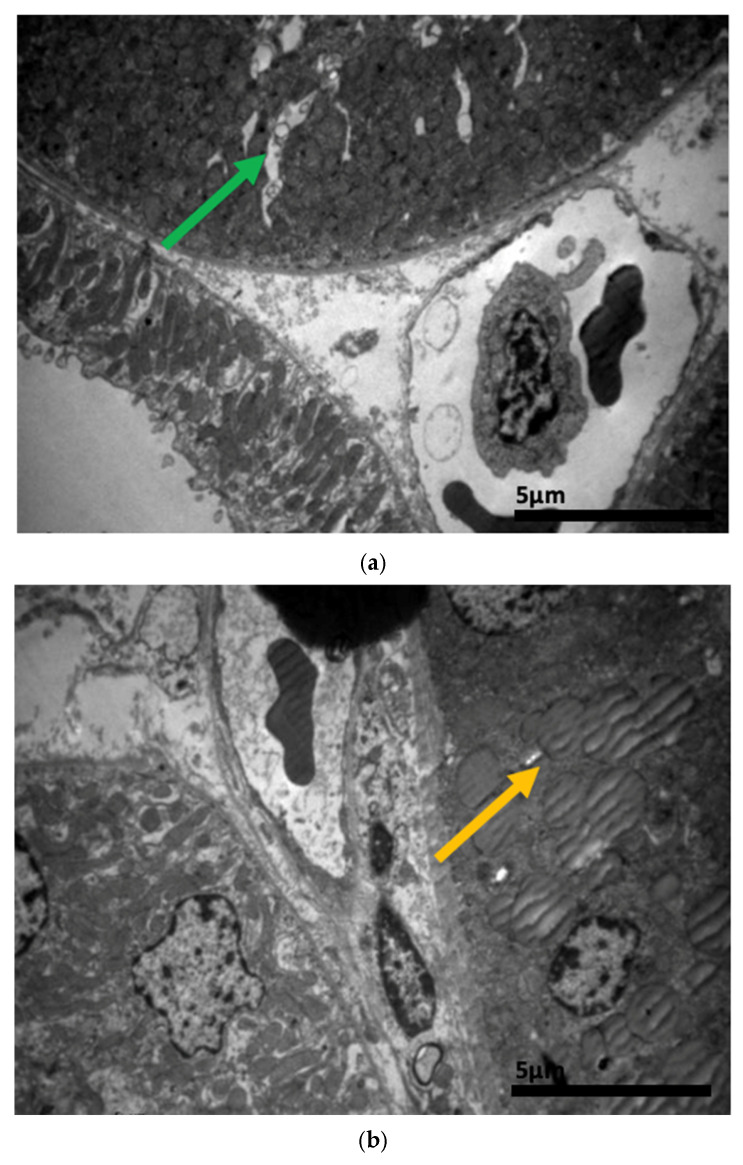

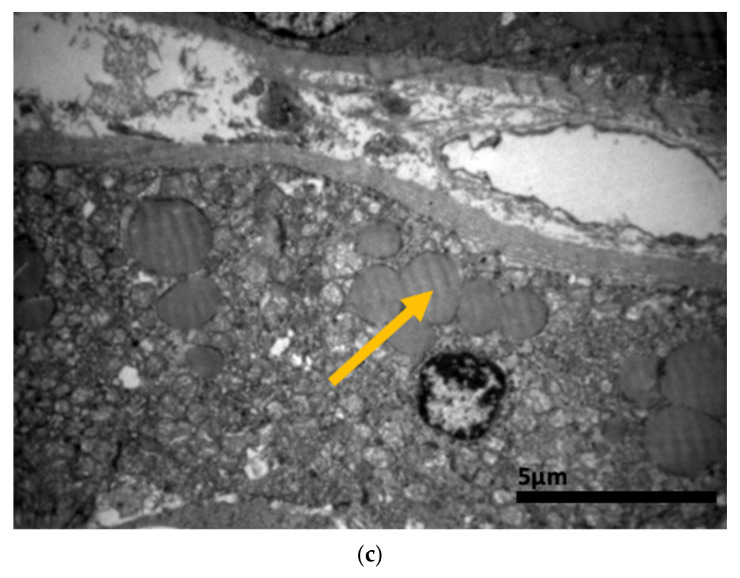
(**a**,**b**,**c**) Following intravitreal administration of a single dose of micafungin, a localized small increase in the intercellular space (↑) in the proximal convoluted tubule is evident, with preservation of the normal histological structure of the distal convoluted tubule. Moreover, following the administration of a single dose of micafungin, the normal cytoarchitecture of the distal convoluted tubule on the left is contrasted again with the locally disturbed one of the proximal convoluted tubules on the right, where the presence of lipid droplets (↑) is evident. ×5000.

**Figure 19:**
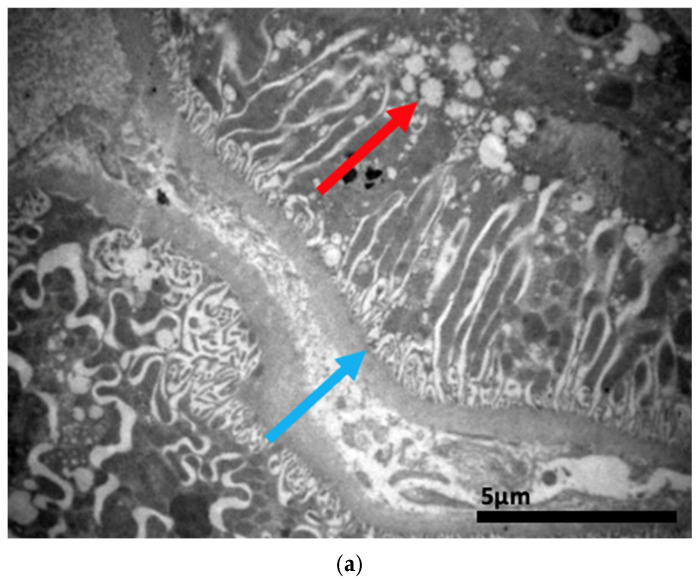

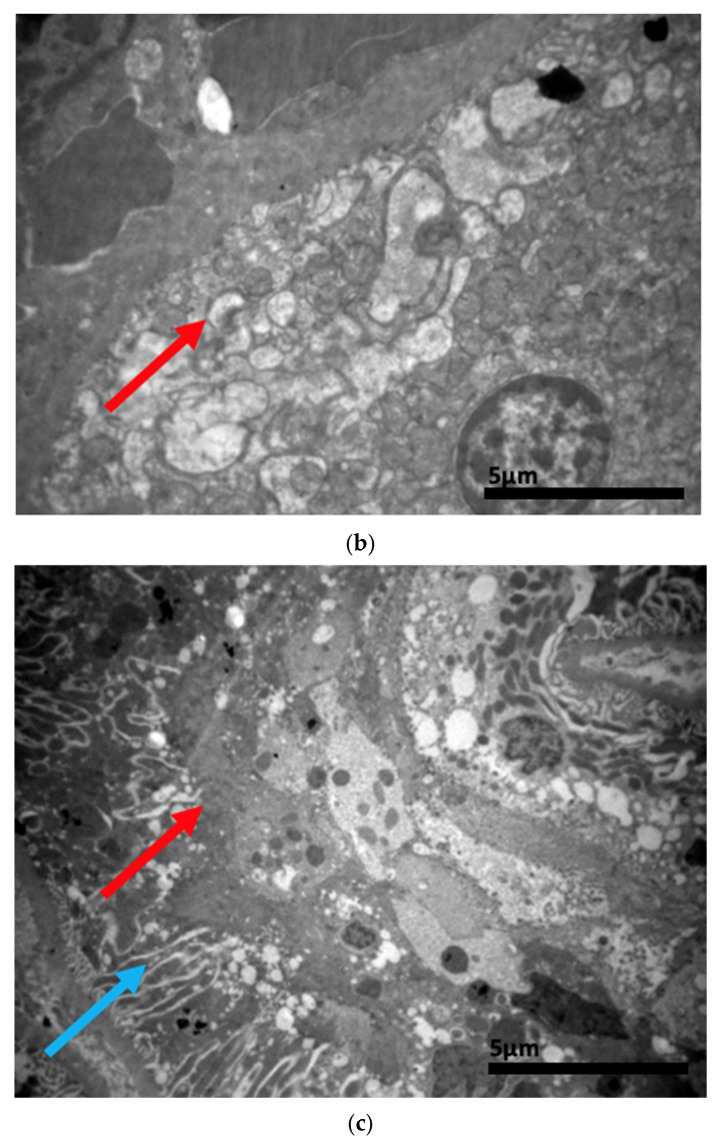
(**a**,**b**,**c**) The administration of two doses of voriconazole resulted in a localized increase along with the widening of the pleats of the basement membrane (↑), together with the presence of vacuoles always at the level of the proximal convoluted tubule. After the injection of two doses of voriconazole, the presence of vacuoles (↑) near the basement membrane of the wall of the renal tubules, with a tendency to coalesce, and the disturbance of the cellular architecture is also confirmed at higher magnification. ×6000 (**a**), ×8000 (**b**), ×4000 (**c**).

**Figure 20:**
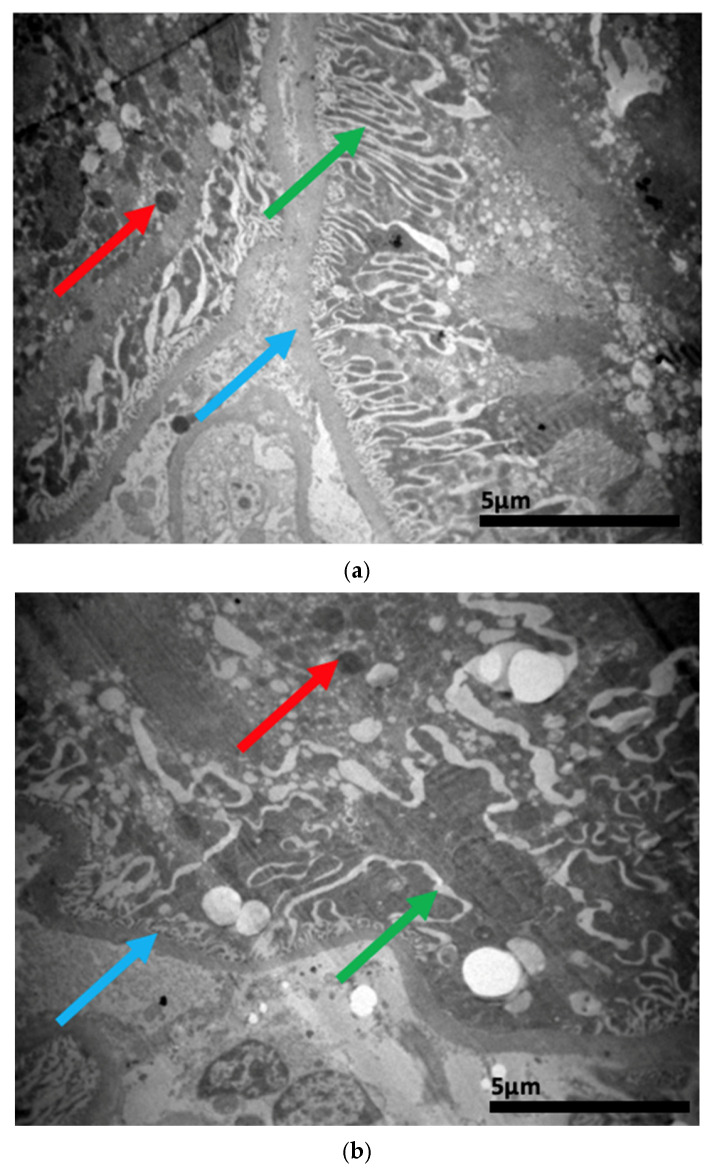
(**a**,**b**) After the administration of two doses of micafungin, more prominent disturbances in the architecture of the cells of the proximal convoluted tubule are observed, characterized by an increase in the intercellular spaces (↑) and a widening of the pleats of the basement membrane (↑). The fusion between the basal membranes of capillaries and tubules is also evident, along with the disappearance of the existing connective tissue between them and the appearance of mitochondria (↑). ×4000 (**a**), ×5000 (**b**).

**Figure 21:**
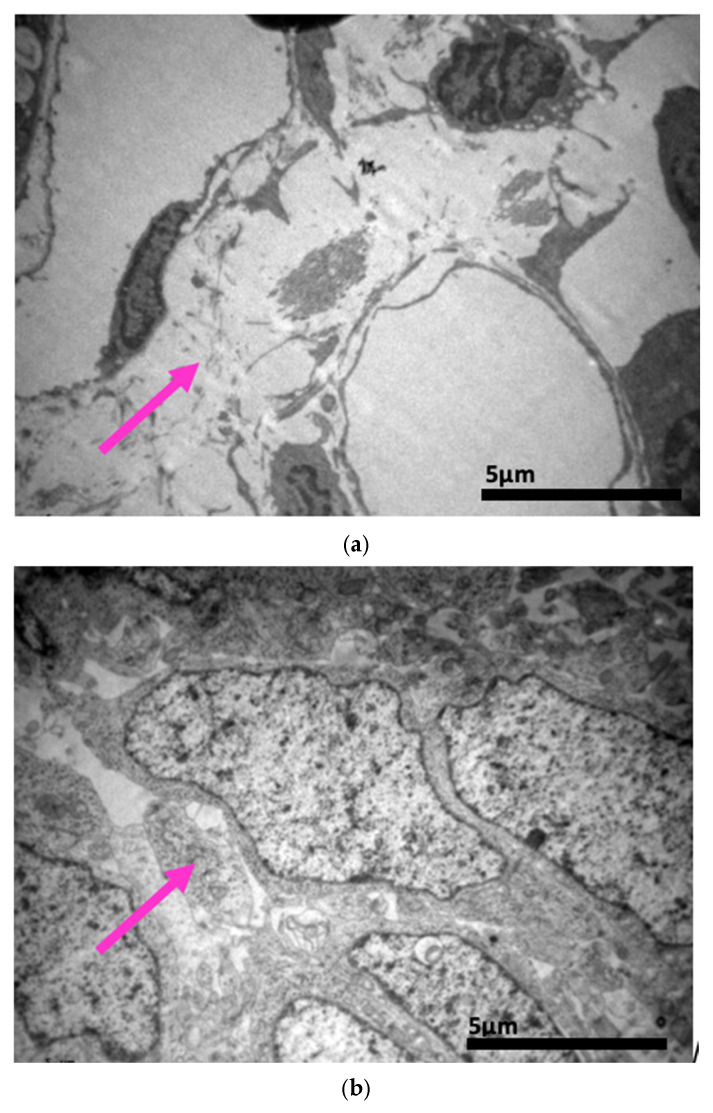
(**a**,**b**) Following the combined intravitreal injection of micafungin and voriconazole, connective tissue edema (↑) occurs in the intercellular spaces. ×5000 (**a**), ×8000 (**b**).

The ultrastructural examination revealed notable changes in renal tissue architecture following the administration of voriconazole and micafungin, either individually or in combination. After a single dose of voriconazole (Figure 17), vacuolar degeneration was observed in the proximal convoluted tubule cells, characterized by the presence of small intracellular vacuoles. Additionally, focal fusion of the podocyte pedicels and enlargement of the Bowman’s capsule urinary space were evident. In contrast, a single intravitreal dose of micafungin (Figure 18) led to a minor, localized increase in intercellular spaces within the proximal convoluted tubules, while the distal tubules maintained a normal histological structure. Some lipid droplets were also identified in the proximal convoluted tubules, indicating mild localized disturbances. More pronounced renal alterations occurred following two doses of voriconazole (Figure 19). These included widening of the basement membrane pleats and increased vacuolization at the level of the proximal convoluted tubules. Vacuoles near the basement membrane showed a tendency to coalesce, and disruption of cellular architecture was evident under higher magnification. Similarly, two doses of micafungin (Figure 20) resulted in increased intercellular space, widening of the basement membrane pleats, and notable structural disruption within the proximal convoluted tubule cells. There was also evidence of fusion between the basal membranes of capillaries and tubules, with loss of intervening connective tissue and the presence of mitochondria within the affected regions. Finally, the combined administration of micafungin and voriconazole (Figure 21) led to connective tissue edema in the intercellular spaces, further indicating an additive or synergistic effect of the two drugs on renal tissue integrity.

## 3. Discussion

Our immunohistochemical findings demonstrate that intravitreal injections of voriconazole and micafungin provoke systemic renal inflammation, evidenced by increased IL-6 and EGFR expression. This observation is consistent with previous reports that inflammatory mediators such as IL-6 contribute to kidney injury and repair mechanisms [117,118], while EGFR activation has been implicated in tubular proliferation and fibrosis in nephrotoxicity models [123,126]. The stronger EGFR activation following voriconazole administration, particularly after repeated dosing, suggests that azoles may preferentially engage EGFR-related pathways, potentially leading to maladaptive repair [123,126]. Conversely, micafungin elicited milder activation of both IL-6 and EGFR, in line with its more favorable renal toxicity profile [80,83,84,85,132]. The synergistic increase in marker expression after combined administration raises concerns about additive renal inflammation, which is clinically relevant given reports of nephrotoxic risk with combination antifungal therapy [127,128,132].

Light microscopic evaluation confirmed that intravitreal administration of voriconazole or micafungin led to disturbances in both renal corpuscles and renal tubules, with changes becoming more pronounced after repeated dosing. Glomerular thickening, enlargement of Bowman’s space, and tubular dilation are alterations typical of early nephrotoxic injury, reflecting mesangial expansion and impaired tubular reabsorption [128,132]. Combination therapy produced the most pronounced disruption, including interstitial edema, glomerular shrinkage, and severe tubular dilation, consistent with cumulative or synergistic effects reported when multiple nephrotoxic agents are used concurrently [127,128,132].

Electron microscopy provided additional evidence of proximal tubular stress and injury. Vacuolar degeneration, basement membrane pleating, and podocyte foot process fusion were observed following voriconazole administration, while micafungin produced localized widening of intercellular spaces and lipid droplet accumulation. These findings are in agreement with previous experimental studies of voriconazole-induced renal lesions [42,105] and with the recognized vulnerability of proximal tubular cells to xenobiotics through transporter overload, mitochondrial dysfunction, and oxidative stress [101,102,103,104,137]. After repeated or combined dosing, fusion of glomerular and tubular basement membranes and connective tissue edema were noted, further supporting the concept of structural barrier disruption and interstitial injury [88,113].

Taken together, these results emphasize that intravitreal administration, although designed to achieve local ocular effects, does not eliminate systemic absorption. Small lipophilic molecules such as voriconazole are known to undergo posterior clearance into the choroidal vasculature, reaching the systemic circulation [89,90,91,92,93,94,95,96,97,98,99]. Systemic consequences of intravitreal therapies have been previously documented, as in the case of anti-VEGF agents, where reduced plasma VEGF activity has been associated with hypertension, proteinuria, and chronic kidney disease [99,100]. Our findings extend this concern to intravitreal antifungals, highlighting the kidney as a target organ for systemic toxicity [131,132].

The clinical implications are noteworthy. Patients receiving repeated or combined intravitreal antifungal therapy, particularly those with pre-existing renal disease or concurrent nephrotoxic drugs, may be at risk of developing renal injury. Careful evaluation of renal function before and during therapy should be considered, along with cautious dosing strategies. The observed upregulation of IL-6 and EGFR also supports the exploration of biomarker-based monitoring approaches to detect early subclinical nephrotoxicity [111,117,118].

This study has limitations. The small number of animals reflects adherence to the principles of the 3Rs (Replacement, Reduction, Refinement) [138,139,140,141,142,143,144]. In addition, the relatively short observation period prevents assessment of the long-term reversibility of the lesions. Functional renal parameters such as serum creatinine or BUN were not assessed, and pharmacokinetic data correlating intravitreal administration with systemic concentrations were not collected. These limitations restrict generalizability but do not diminish the mechanistic insights obtained.

In summary, the present study provides novel evidence that intravitreal administration of voriconazole and micafungin results in systemic renal alterations detectable at histological, ultrastructural, and molecular levels. Voriconazole produced stronger nephrotoxic changes than micafungin, and their combined use led to synergistic damage. These findings underscore the need to balance the ocular benefits of intravitreal antifungal therapy against potential systemic risks and support further studies on optimized dosing, pharmacokinetics, and renal monitoring. Future research priorities include pharmacokinetic studies of systemic absorption, longitudinal renal function follow-up, and interventional studies evaluating lower doses or alternative antifungals like lactate-stabilized formulations to help optimize intravitreal therapy while minimizing renal exposure.

## 4. Materials and Methods

### 4.1. Selected Animals

Τhe laboratory animals employed in the experimental procedure of the present study were the New Zealand white rabbits. The selected number of animals was six (6), meeting the specifications of the 3Rs (Replacement, Reduction, and Improvement), aiming to apply to the animals the most acceptable study conditions possible. Moreover, the rabbits were five (5) months old and weighed two to four (2–4) kilograms, including both male and female animals.

Before the experimental procedure was carried out, a research protocol permit was granted by the “Bioethics and Ethics Committee” of the Medical Department of the School of Health Sciences of the Aristotle University of Thessaloniki, having the number 4.209/17-7-19, as well as a research protocol permit from the “Directorate of Veterinary 66 Medicine” of the Greek Ministry of Health, Department of Animal Health and Veterinary Perception, Medicines and Applications (YZ-KAFE) with protocol number 57253(211) on 15 February 2019.

The selection of these specific laboratory animals was based on the extensive international literature, according to which New Zealand white albino rabbits are perfectly suitable for the present experimental intervention [138,139,140,141,142,143,144]. The primary reason that justifies the above choice is the eye anatomy and histology of the rabbits, which simulates that of the human sense organ, while their size, which is larger compared to other animals, rats, for instance, facilitates the application of the experimental protocol. Additionally, rabbits’ liver and kidneys also demonstrate significant similarities to the corresponding human organs.

The final decision for a satisfactory sample size, representative of the results, was not based on pre-existing studies, as there are no publications in the international literature regarding the expression of the peculiar pro-inflammatory cytokines examined in the current investigation in the eyes, the liver, or the kidneys after voriconazole or micafungin administration. For the above reason, the selection of the number of animals was based on fulfilling the requirements of the aforementioned 3Rs. This condition ensures that the introduction of the smallest possible number into the experimental procedure will not affect the outcome of the research study while ensuring that the animals are subjected to the least possible suffering. Furthermore, in an effort to mobilize the smallest possible number of laboratory animals, both eyes of each rabbit were deployed, the right as part of the study group, and the left as a constituent of the control group. The animals’ living conditions met all the necessary criteria for their well-being. They were kept in cages made of friendly raw materials, ergonomically designed to provide proper ventilation supplied by an air duct system, maintaining constant temperature and humidity conditions, proper feeding and care, i.e., daily supply of clean water, concentrated food, and replacement of the hay. Last but not least, the basic equipment of the cages included a lightning regulation system [145,146,147,148,149].

### 4.2. Forming Groups and Subgroups

In the course of the experimental procedure, the animals’ eyes were classified into the study groups V (voriconazole administration), M (micafungin administration), and VM (voriconazole and micafungin co-administration), as well as and the control group C. The group V included six rabbits administered a single or double intravitreal administration of voriconazole, the group M included six rabbits that received a single or double intravitreal injection of micafungin, the group VM included three rabbits in which co-administration of both antifungals was performed, and ultimately, the group C consisted of the three rabbits that received no medication. In more detail, the established groups and subgroups were the following [150,151]:

Control group C: consisting of 3 rabbits that were not given any medication in order to study the kidney, since the above drugs are systemically metabolized

Group V: consisting of six (6) rabbits and subdivided into: Subgroup V1: consisting of three (3) rabbits that received one (1) intravitreal injection of voriconazole solution in their right eyes and one (1) intravitreal administration of Balanced Salt Solution (BSS) in their left eyes on day 0, Subgroup V2: also consisting of three (3) rabbits in which two (2) intravitreal injections of voriconazole solution were performed in their right eyes and two (2) intravitreal injections of BSS in their left eyes on days 0 and 4.

Group M: consisting of six (6) rabbits and subdivided into: Subgroup M1: consisting of three (3) rabbits that received one (1) intravitreal injection of micafungin solution in their right eyes and one (1) intravitreal administration of BSS in their left eyes on day 0, Subgroup M2: also consisting of three (3) rabbits in which two (2) intravitreal injections of micafungin solution were performed in their right eyes and two (2) intravitreal injections of BSS in their left eyes on days 0 and 4.

Group VM: consisting of three (3) rabbits in which one (1) intravitreal injection of voriconazole solution was performed in their right eyes on day 0 and one (1) intravitreal injection of micafungin solution was performed in their right eyes on day 4, while two (2) intravitreal injections of BSS were performed in their left eyes on days 0 and 4.

### 4.3. Experimental Protocol

As far as the days when the injections were taken place are concerned, for the subgroups V1, M1, the first injection was performed on day 0, followed by euthanasia on day 10, whilst for the subgroups V2, M2, VM, the first injection was performed on day 0, the second on day 4, followed by the euthanasia on day 14. During the experimental procedure, solutions of voriconazole (VFend, Pfizer Europe MA EEIG), micafungin (Mycamine, Astellas Pharma Europe B.V.), or normal saline Balanced Salt Solution (BSS) (IOLART, Greece) were injected into the rabbits’ eyes’ medial vitreous. The dosage of the solutions was adjusted based on the volume of the rabbits’ vitreous, which was, on average, calculated to be 1.6 mL. This adjustment determined the dose of voriconazole at 40 μg/0.1 mL, micafungin at 25 μg/0.1 mL, and BSS at 0.1 mL. Table 4 summarizes medication doses for each group. More precisely, in the treatment of fungal endophthalmitis, the standard maximum intravitreal concentration in the human eye is 100 μg. In rabbits, where the intravitreal concentration is limited to 1/4 of the aforementioned, there is a demand for a reduction of this concentration, which resulted in the above-selected doses of the particular antifungal agents. Moreover, according to studies, the intravitreal concentration of 25 μg/0.1 mL is the safe limit for the retina, up to which no lesions were observed using an optical microscope. Typical intravitreal doses for antifungals like voriconazole in humans are around 100 µg/0.1 mL [129], while monoclonal antibodies such as anti-VEGF agents are administered at doses ranging from 0.5 mg to 2 mg per injection in humans [152]. In our study, intravitreal dosing in rabbits was scaled according to the differences in vitreous volume between species (rabbit vitreous volume approximately 1.5 mL versus human approximately 4 mL), with doses applied approximately proportionally to achieve comparable intraocular concentrations. This dosing strategy is consistent with established preclinical pharmacokinetic studies that aim to achieve clinically relevant ocular exposure levels [133,136]. A typical dose administered of voriconazole is 200 mg orally or intravenously twice daily for systemic fungal infections [131]. Nephrotoxicity is reported at prolonged high doses or in patients with pre-existing kidney impairment [128,132]. Renal adverse effects, including acute kidney injury and proteinuria, have been documented in some cases [128]. Reports indicate that nephrotoxicity is dose- and exposure-dependent, often emerging after weeks to months of systemic therapy [127,128]. Before the administration of the solutions, rabbits were weighed so that their exact weight could be ascertained. Intravitreal administration was selected, since it is considered a local route of drug delivery, specifically designed to maximize therapeutic concentration at the site of ocular infection, such as in fungal endophthalmitis, while minimizing systemic exposure. Intravitreal injection is required to achieve therapeutic intraocular drug levels, as systemic administration results in very limited ocular penetration. The blood-retinal barrier restricts drug entry, with intravitreal concentrations of voriconazole typically less than 1% of plasma levels after oral or intravenous dosing [129]. Similarly, large molecules like monoclonal antibodies show negligible intraocular exposure following systemic delivery [152]. Thus, systemic routes cannot replace local intravitreal administration [129,131]. After intravitreal injection, drugs are cleared in two phases, lasting from hours to days, which helps maintain effective levels inside the eye while keeping systemic exposure low. In contrast, oral or intravenous doses quickly reach peak levels in the blood, but only a small amount reaches the eye because of the blood-retinal barrier. This highlights why direct intravitreal injection is necessary to achieve effective treatment in the eye with minimal impact on the rest of the body [129,131,133,136]. Intravitreal injection was followed by the procedure of preanesthesia performed by intramuscular (i.m.) administration of butorphanol 0.1 mg/kg and dexmedetomidine 50 μg/kg, and the general anesthesia was accomplished with intramuscular (i.m.) injection of ketamine at a dose of 25 mg/kg and intravenous (i.v.) administration of propofol 0.5 mg/kg. After that, local anesthesia of the ocular surface with 0.5% proparacaine hydrochloride and pupillary mydriasis with 1% tropicamide were conducted. Intravitreal injection of the voriconazole, micafungin, and BSS solutions with a 30-gauge needle was performed in the center of the vitreous, followed by the placement of a cotton patch at the injection site for the prevention of any possible leakage of the administered solutions from the injection site. Euthanasia, after the ophthalmic examination of the animals by slit-lamp and indirect ophthalmoscopy, was performed on day 10, following the last intravitreal injection, and was accomplished by the administration of a combination of dexmedetomidine, ketamine, propofol, and potassium chloride. Finally, the retina, liver, and kidneys of the animals were removed and sampled. This was followed by the preparation of the kidney samples for examination by optical microscopy, immunohistochemistry, and electron microscopy, while the liver samples were kept for future analysis.

Due to the fact that the elimination routes of these antifungal drugs employ the kidneys as part of the excretory system, it was of pivotal importance to investigate the probability of nephrotoxicity onset after the intravitreal administration of these agents [137,151,152,153,154,155,156,157,158].

The dosage of the solutions was adjusted based on the volume of the rabbits’ vitreous, which was, on average, calculated to be 1.6 mL.

### 4.4. Sample Preparation Prior to Optical Microscopy

Initially, the samples were cut into tissue pieces having a thickness of 0.5–4.0 cm and then were enclosed in cassettes. For their fixation, a 10% formalin solution prepared from a 35% formaldehyde solution was elaborated. Dehydration was carried out in ascending order of alcohols 76%, 96%, 100%, and 100%. Hereafter, the samples were cleaned with xylene solvent for four (4) hours. Tissue pieces were then cut into sections of three (3) μm of thickness using a semi-automatic microtome. From these, ten (10) sagittal sections were chosen by systematic random sampling methodology, and three (3) of them were placed on microscope slides, while the remaining seven (7) were placed on positively (+) charged plates. To remove the excess water, plates were first left at room temperature for one (1) hour, then placed in a 65 °C oven for one (1) hour, and finally immersed in a xylene solution for ten (10) minutes. They were then treated with a descending series of alcohol solutions: 100%, 100%, 96%, and 76%. Staining was firstly applied with hematoxylin solution for five (5) minutes, followed by washing with tap water for five (5) minutes and partial decolorization of the stain with 1% contrast solution for one (1) sec. This was followed by eosin solution staining for one (1) min, followed by dehydration in ethanol for five (5) min, and processing in xylene solution for five (5) min. Hindermost, just before microscopy, the slides were coated with Canada balsam.

### 4.5. Sample Preparation Prior to Immunohistochemistry

Immunohistochemical staining was then performed, in an attempt to spotlight inflammation signs. The histological samples’ preparation prior to the application of the immunohistochemical techniques was conducted by the following procedure. Thin sections were taken from the paraffin-embedded tissue and remained overnight in the oven. The Envision Flex Kit was deployed for the procession of the sections, according to the listed instructions. At the beginning of the procedure, deparaffinization took place, in which the sections were treated with xylene solution 2 times for 10 min each time. The samples were then immersed in a descending order of absolute ethanol 96%, 80%, and 70% and the process was repeated three (3) times for ten (10) minutes each time. The sections were later washed with distilled water. Following this, samples were treated with Antigen Retrieval Fluid and left for 20 min in a microwave oven 450 W in order to reveal the epitopes; the antigenic determinants that are often masked during the fixation process. The sections were afterwards washed with phosphate-buffered saline (PBS) and incubated for 10 min with hydrogen peroxide to suppress the activity of the endogenous peroxidase (Peroxidase Blocking Reagent), in an effort to avoid any unwanted staining, and then were washed again with PBS. Following this, the application of the primary antibody solution was conducted in order to examine its potential binding to the corresponding tissue antigen. The two primary antibodies used were IL-6 (dilution 1:100) and EGFR (dilution 1:100). Afterwards, the samples were washed with water. The sections were then cleared in Envision Wash Buffer for ten (10) minutes. For the detection of the immunohistochemical staining, the samples were immersed in the secondary antibody polymer solution that binds to the primary antibody, then washed, and treated with the chromogen: 3,3-diaminobenzidine solution for five (5) minutes. This was followed by the washing of the samples with tap and distilled water. At a later time, the sections were stained with hematoxylin solution and then washed again with tap and distilled water. This was followed by the samples’ drying in an ascending order of alcohols 70%, 80%, and 96% three times, for 10 min each time, and the treatment with xylene solution twice, for 10 min each time. Finally, before their microscopic examination, the sections were fixed on a plate with a coverslip (mounting). Positively charged slides were used for an immunohistochemical study, after adding the EGFR and anti-IL-6 antibodies and observing them under bright field microscopy by at least two (2) independent observers, blinded to the identity of the immunohistochemical preparations. For the quantification of the results, the intensity of the immunohistochemical staining was observed based on a semiquantitative cross-scale, which included the following grades: negative/no staining; no cross (–), mild staining; one cross (+), moderate staining; two crosses (++), intense staining; three crosses (+++).

### 4.6. Statistical Analysis

Immunohistochemical staining was evaluated using the ImmunoReactive Score (IRS) method, which combines staining intensity and the percentage of positive cells to generate a semi-quantitative score. Staining intensity was scored on a scale from 0 to 3 (0 = no staining, 1 = weak, 2 = moderate, 3 = strong), and the percentage of positively stained cells was scored from 0 to 4 (0 = <5%, 1 = 5–25%, 2 = 26–50%, 3 = 51–75%, 4 = >75%). The final IRS was calculated by multiplying the intensity and percentage scores, yielding a total score ranging from 0 to 12. Scoring was performed manually by two independent observers to ensure consistency.

### 4.7. Sample Preparation Prior to Electron Microscopy

First and foremost, the kidney tissue was cut into pieces smaller than one cubic centimeter (<1 cm^3^). This was followed by the fixation of the pieces, which was driven by immersing them in a 3% glutaraldehyde solution for two (2) hours, rinsing them in a phosphate buffer solution for ten (10) minutes, and postfixing them in a 1% osmium tetroxide (OsO4) solution for one and a half (1½) hours. Afterwards, an intermediate washing was performed firstly with phosphate buffer solution for ten (10) minutes and then with bi-distilled water for another ten (10) minutes. The tissues were stained parallel to their fixation with 1% uranyl acetate solution for sixteen (16) hours. The removal of the excess water was achieved by progressive dehydration with an ascending series of alcohols: 30° for five (5) minutes, 50° for five (5) minutes, 70° for five (5) minutes, 96° for five (5) minutes, and 100° for five (5) minutes, 6 times. Ultimately, the samples were immersed in Epon resin, and very thin sections of 60–90 nm were taken employing a microtome. Before the final observation in a TEM JEOL 1011 electron microscope at 80 kV (JEOL-Tokyo, Tokyo, Japan), the sections were stained with Reynold’s stain.

### 4.8. Study Advantages-Uniqueness

To our knowledge, there are no similar histological and immunohistochemical studies available in the international literature on the effect of the antifungal agents voriconazole and micafungin on renal tissue, after intravitreal administration of the specific drugs, determining their possible toxic side effects as a consequence of the application of the maximum safe dose.

### 4.9. Study Limitations

The limitations of the present study could involve initially the limited laboratory animals’ number, which was selected as mentioned above, with the ultimate goal of introducing the smallest possible sample size into the experimental protocol. Furthermore, it must be emphasized that all the beyond alterations were observed within a period of ten (10) days from the last intravitreal administration of the applied antifungal agents, which may be considered as a short period of time for the observation of the reversibility or not of the inspected lesions. The above limitations are considered by all means an opportunity for further expansion of the present investigation in the future.

## 5. Conclusions

In conclusion, after the intravitreal administration of two antifungal agents: an azole, voriconazole, and an echinocandin, micafungin, both microscopic and ultramicroscopic lesions of the renal tissue were observed. The presence of these lesions substantiates the observation that the intravitreal route of administration of pharmaceutical preparations, although it is by definition thought to be a local route of administration, nevertheless leads to a certain percentage of systemic absorption of the administered agents. It is also an important indication of the participation of the kidney in the processes of metabolism and excretion of the specific antifungal drugs after this intravitreal injection.

Dividing the possible effect of the two drugs on the renal tissue, it could be said that the lesions as revealed mainly through the immunohistochemical staining of the preparations showed a rather greater intensity in the case of voriconazole administration, an observation possibly attributed to the more favorable toxicological profile of micafungin that as an echinocandin affects kidney function to a lesser extent. In addition, regarding the outcome of the dose on the appearance of the toxic effects of these antifungal drugs on the renal structure and organization, it appears that, both from this study via light and electron microscopy, the lesions were more pronounced with the application of a second dose of the same agent. Lastly, the simultaneous administration of the two drugs led to more prominent histological lesions.

Whatsoever, the appearance of these changes in places and to a lesser extent than in the case in which these drugs would be administered systematically raises the question of the possible need to reduce the dose in order to achieve a lower intravitreal concentration in the case of their administration during the treatment of fungal endophthalmitis. From this perspective, it is also necessary to study the patient’s medical history by examining his renal function before the intravitreal injection of the specific agents, while monitoring renal function throughout the treatment. Despite the aforementioned findings, the effort to reveal the possible toxic effects of these drugs will certainly require further research and study in an effort to understand the mechanisms behind the possible damage to the kidney tissue.

## Figures and Tables

**Table 1:** Administered drug properties.

Antifungal Agent	Structure	Metabolism
Voriconazole	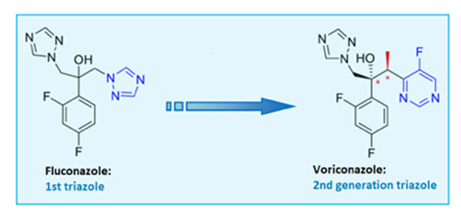	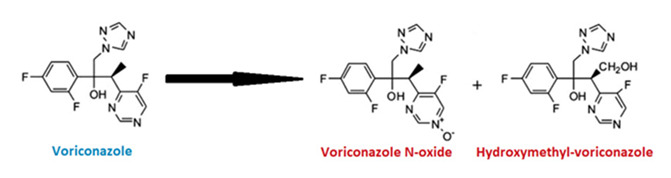
Micafungin	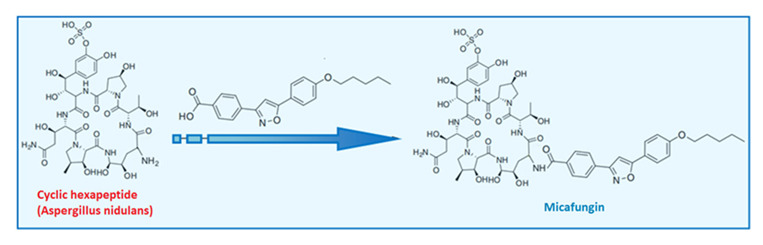	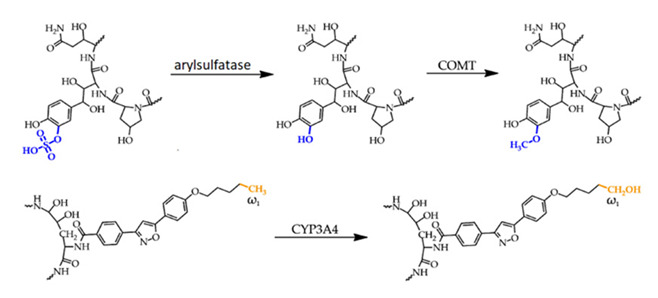

**Table 2:** Results of IRS.

Subgroup	Structure	Biomarker	Intensity	Positively Stained Cells	IRS
C	Renal tubules and corpuscles	IL-6	1	2	2
C	Renal tubules and corpuscles	EGFR	1	1	1
V1	Renal tubules	IL-6	2	4	8
V1	Renal corpuscles	IL-6	2	2	4
V1	Renal tubules	EGFR	3	4	12
V1	Renal corpuscles	EGFR	2	2	4
V2	Renal corpuscles	IL-6	2	1	2
V2	Renal tubules	IL-6	2	3	6
V2	Renal corpuscles	EGFR	3	4	12
V2	Renal tubules	EGFR	2	4	8
M1	Renal corpuscles	IL-6	2	3	6
M1	Renal tubules	IL-6	2	1	2
M1	Renal corpuscles	EGFR	3	4	12
M1	Renal tubules	EGFR	1	2	2
M2	Renal corpuscles	IL-6	3	3	9
M2	Renal tubules	IL-6	2	3	6
M2	Renal corpuscles	EGFR	1	2	2
M2	Renal tubules	EGFR	1	3	3
VM	Renal corpuscles	IL-6	3	4	12
VM	Renal tubules	IL-6	1	2	2
VM	Renal corpuscles	EGFR	3	3	9
VM	Renal tubules	EGFR	2	3	6

**Table 3:** Immunohistochemical expression of IL-6 and EGFR in renal tissue (IRS values) in different experimental groups.

Marker	Tissue	Control (C)	Voriconazole (V)	Micafungin (M)	Voriconazole + Micafungin (VM)	*p*-Value	Significant Comparisons (Dunn’s Post Hoc)
IL-6	Renal Tubules	2 (1–4)	3 (1–5)	3 (2–5)	5 (3–6)	0.048	VM > C (*p* = 0.03)
	Renal Corpuscles	1 (0–3)	2 (1–4)	4 (2–5)	5 (3–6)	0.0089	VM > C (*p* = 0.006), M > C (*p* = 0.049)
EGFR	Renal Tubules	3 (2–4)	5 (3–6)	4 (3–6)	6 (5–7)	0.0197	VM > C (*p* = 0.012), V > C (*p* = 0.043)
	Renal Corpuscles	3 (2–4)	4 (3–5)	5 (4–6)	6 (5–7)	0.0247	VM > C (*p* = 0.013), M > C (*p* = 0.038)

**Table 4:** Summarizing the medication and the dose administered to each group.

Animal Group	Medication Administrated	Dose Applied
Control group C	No medication administrated	-
Study Subgroup V1	One (1) intravitreal injection of voriconazole solution	40 μg/0.1 mL
Study Subgroup V2	Two (2) intravitreal injections of voriconazole solution	40 μg/0.1 mL (each injection)
Study Subgroup M1	One (1) intravitreal injection of micafungin solution	25 μg/0.1 mL
Study Subgroup M2	Two (2) intravitreal injections of micafungin solution	25 μg/0.1 mL (each injection)
Study group VM	One (1) intravitreal injection of voriconazole solution and one (1) intravitreal injection of micafungin simultaneously	40 μg/0.1 mL (voriconazole) 25 μg/0.1 mL (micafungin)

## Data Availability

The original contributions presented in this study are included in the article. Further inquiries can be directed to the corresponding authors.
